# Excess cost of non-remission among outpatients with major depressive disorder

**DOI:** 10.1186/1752-4458-8-42

**Published:** 2014-11-15

**Authors:** Jong-Min Woo, Hong Jin Jeon, Hyo-Jin Kim, Kwang-Hun Lee, Chang Uk Lee, Jai Sung Noh, Chang Hwa Lee, Jin Pyo Hong

**Affiliations:** Department of Psychiatry, Seoul Paik Hospital, Inje University School of Medicine, Seoul, Korea; Stress Research Institute, Inje University, Seoul, Korea; Department of Psychiatry, Depression Center, Samsung Medical Center, Sungkyunkwan University School of Medicine, Seoul, Korea; Depression Clinical and Research Program, Massachusetts General Hospital, Harvard Medical School, Boston, USA; OR/EBM Team, Market Access Division, Pfizer Pharmaceuticals Korea Limited, Seoul, Korea; Department of Psychiatry, Dongguk University College of Medicine, Gyeongju, Korea; Department of Psychiatry, College of Medicine, The Catholic University of Korea, Seoul, Korea; Department of Psychiatry, Ajou University School of Medicine, Suwon, Korea; Department of Psychiatry, Eulji University School of Medicine, Daejeon, Korea; Department of Psychiatry, Asan Medical Center, University of Ulsan College of Medicine, Seoul, South Korea

**Keywords:** Major depressive disorder, HPQ, Lost productive time, Presenteeism, Remission

## Abstract

**Background:**

The purpose of this study was to assess the economic benefit of achieving remission among outpatients with major depressive disorder (MDD) who are currently employed in Korea.

**Methods:**

Cross-sectional observational study. A total of 337 outpatients with MDD with paid jobs were recruited from 14 psychiatric clinics in Korea and were then divided into three groups as follows: new visit group (n = 128), remitted group (n = 100) and non-remitted group (n = 109). The 17-item Hamilton Depression Rating Scale (HAM-D) was used to decide whether a patient should be assigned to the remitted or non-remitted group. Direct medical and non-medical costs were measured via interview with the subjects. The World Health Organization Health and Work Performance Questionnaire (HPQ) were applied in order to measure the lost productive time (LPT) and related productivity costs.

**Results:**

The three groups did not show a significant difference in direct medical cost. However, the difference between the remitted group and non-remitted group was statistically significant (25.49 ± 52.99 vs. 44.79 ± 126.55, *χ*^2^ = 12.99, p = 0.0015). The remitted group demonstrated a significant improvement in productivity (particularly presenteeism) when compared with the new visit group (Z = −3.29, p = 0.001). Although the non-remitted group received treatment at psychiatric clinics similar to the remitted group, it lost 33 more working hours per month, which is compatible to $332 per month.

**Conclusion:**

These results suggest the economic importance of achieving remission in treating depression.

## Background

The economic burden from depression can be assessed by measuring direct costs, including medical and non-medical expenses (i.e., transportation) and productivity costs, particularly focusing on the wage loss at work. Productivity costs can be higher than direct costs in mental health problems, even though the relative composition can be varied by the economy and health insurance coverage of each society [1, 2]. Major Depressive Disorder (MDD) is one of the major mental health conditions among adults and has been shown to have a large impact on work productivity [3, 4]. The treatment of depression has been shown to increase productivity as well as have a favorable return-on-investment (ROI) [5–8].

When measuring productivity cost, many studies use the concept of “lost productive time” (LPT), which consists of “absenteeism” and “presenteeism.” Absenteeism refers to the LPT caused by hours or days missed from work (e.g., tardiness, leaving work early, sick leave) [2]. Presenteeism is defined as the estimated LPT caused by reduced work performance while at work [9], which can result from impaired concentration, reduced motivation, fatigue or errors in decision-making. For depressive disorders, LPT from presenteeism has been shown to exceed LPT from absenteeism, according to a recent study performed in Korea [4].

Measuring both economic loss and work productivity is very important for depressed patients who work for pay, as they may be unable to maintain employment if their productivity is severely impaired. The goal of treatment for depression can vary depending on the patient’s status. For depressed workers, returning their work performance to the maximum level might be the most critical target of receiving treatment. Improved productivity is associated with stabilizing one’s basic condition of social life, maintaining self-esteem and even getting back one’s purpose of living.

In this sense, it is quite obvious to say that getting better is not enough. Many studies have consistently suggested the importance of achieving remission, which is defined as almost full recovery to the level of a person without depression, thereby demonstrating low scores on the symptom severity scale. In contrast to remission, ‘response’ means improvement of 50% or more in depressive symptoms on a rating scale. Depressed patients who failed to obtain full remission portrayed a higher risk of being depressed again [10] as well as continued to experience functional impairment and even brain injury [11]. Zimmerman et al. suggested three factors in patient self-assessment of remission: the presence of features of positive mental health (i.e., optimism, vigor, self-confidence); a return to one’s normal self; and a return to the usual level of functioning [12]. Restoring job functioning is a key component of becoming optimistic, self-confident and being one’s own self.

Although there are various types of antidepressants which can be prescribed, not all patients who receive treatment can achieve full symptom remission and regain their original level of work performance. Furthermore, the social stigma against depression and making visits to a psychiatrist can hinder treatment compliance and remission achievement [13]. Thus, understanding the benefits of gaining remission in reducing LPT would be very important for maximizing the functional outcome.

In this paper, we estimated both the direct costs and indirect costs during the previous month. Regarding direct costs, we gathered information on outpatient medical cost (cost of psychiatric treatment and number of outpatient visit during the past month), transportation expenses, and the frequency and cost of receiving supplementary therapy for treating depression. Regarding indirect costs, we selected data for patients with a paid job and measured the loss of productivity among MDD patients using the World Health Organization’s Health and Work Performance Questionnaire (HPQ) [14]; we then compared it among the three subgroups (new visit, remitted and non-remitted group). We hypothesized that the non-remitted group shows more severe productivity loss than the new visit group and remitted group; further, the severity of specific subtypes of symptoms can be associated with productivity loss.

## Method

### Subjects

The data of Korean MDD patients were derived from the Korean Burden of Illness Study. This is a cross-sectional, multicenter-based, naturalistic and outpatients-based study for MDD. All patients were enrolled from 14 regional psychiatric outpatient clinics in Korea. The study was conducted between December 2011 and September 2012. All study procedures were approved by the Institutional Review Board of the Asan Medical Center and each site. Institutional review board–approved written informed consent was obtained from all study patients before commencing the study.

The regional centers included three sites in the South districts of Seoul: 1) Samsung Medical Center, Sungkyunkwan University School of Medicine, 2) Asan Medical Center, University of Ulsan College of Medicine, and 3) Seoul St. Mary’s Hospital, Catholic University College of Medicine, three sites in the North district of Seoul; 4) Seoul Paik Hospital, Inje University 5) Konkuk University Medical Center, and 6) Kyung Hee University Medical Center, two sites in Incheon; 7) Ajou University Hospital, and 8) Gil Medical Center, Gachon University, two sites in Daejeon; 9) Chungnam National University Hospital and 10) Eulji University Hospital, one site in Cheonan; 11) Dankook University Hospital, one site in Daegu; 12) Kyungpook National University Hospital, 13) one site in Busan; Dong-a University Medical Center, and one site in Gyeongju; 14) Dongguk University Gyeongju Hospital.

### Inclusion and exclusion criteria

The inclusion criteria were those aged between 18 to 64 years with a primary diagnosis of major depressive disorder, single or recurrent non-psychotic, according to the DSM-IV-TR criteria confirmed through clinical diagnosis by psychiatrists. The 17-item Hamilton Depression Rating Scale (HAM-D) was applied to the patients [15]. A total 811 patients were enrolled from 14 hospitals. Each consecutive patient was evaluated according to the inclusion and exclusion criteria and then, allocated to one of three groups -- 1) New visit group, 2) Remitted group (HAMD-17 < 8) or 3) Non-remitted group (HAMD-17 ≥ 8). Each hospital was allowed to allocate about 8 patients to each group, consecutively. Groups 2 and 3 included patients who have received treatment for MDD for less than 6 months in order to exclude the possibility of bias in calculating the lost productivity cost from decreased social occupational function related with other emotional problems.

The exclusion criteria were history of bipolar disorder, schizophrenia, schizoaffective disorder, psychosis, anorexia or bulimia nervosa, obsessive-compulsive disorder, or a serious general medical condition.

### Measure of depressive symptoms

We used the Hamilton Rating Scales for Depression (HAM-D) to measure the severity of depressive symptoms at each visit [16].

### Measure of somatic symptoms

The Depression and Somatic Symptoms Scale (DSSS) was applied in order to measure the somatic symptoms of MDD patients [17]. It is a 22-item self-administered rating scale, including three subscales: Depression Subscale (DS), Pain Subscale (PS) and Somatic Subscale (SS). The DS had 12 items, including three vegetative symptoms and fatigue, whereas the SS had 10 items, including five pain items comprised of the 5-item PS. Each item was rated with a score of 0–3: 0 (not at all); 1 (mild); 2 (moderate); 3 (severe). The range of the sum score is thus 0–66. The scale had good validity and reliability; higher scores demonstrated heavier symptoms [18]. The Korean Version of DSSS had a Cronbach's alpha of 0.90 and showed a relatively high test-retest reliability (r = 0.83, p <0.01) [19].

### Measure of lost productive time (LPT)

We applied the Korean version of HPQ for the measurement of productivity loss. The Korean version of the HPQ was developed using conventional techniques of translation and back-translation by bilingual psychiatrists, maintaining equivalence with the permission of Dr. Kessler, the original author [20].

Most of the HPQ questions ask about the status of the most recent four-week period. In HPQ, absenteeism is the sum of the number of absent work days multiplied by 8 hours a day and the hours absent from partial day absences, such as being late and leaving early from work. Absent work days due to health problems or any other reason are separately assessed.

Presenteeism is measured by the self-rated level of job performance, a visual analogue scale from 0 (worst) to 10 (best), during the past 4 weeks. To ensure a precise appraisal of presenteeism, the HPQ asks participants for an estimate of colleagues’ average productivity as well as for their own average productivity in the past year or two.

Excess lost productive time from non-remission was calculated as the difference between the average LPT in the remission group and the average LPT in the non-remission group.

### Calculation of indirect cost from LPT due to absenteeism and presenteeism

The monthly cost of LPT due to absenteeism is calculated by projecting the total hours missed from work due to health problems over the previous month multiplied by the standard labor hourly wage. Standard labor hourly wage was imputed from the Survey on Labor Conditions by Employment Type (2010) by Korea Statistical Information Service, considering age, gender and academic accomplishment level [21]. LPT due to presenteeism is quantified as the actual work hours multiplied by the reduced performance level (10-productivity rating) over 10. The monthly cost of LPT due to presenteeism was calculated as the total hours lost from reduced performance for the previous month multiplied by the standard labor hourly wage.

### Statistical analysis

Descriptive statistics methods (mean, median and standard deviation) were used for continuous data in MDD patients. Frequency (N) and percentage (%) were obtained for categorical data and the chi-square test was applied in order to compare these categorical data.

ANOVA (or Kruskal-Wallis test in case of nonparametric data) was performed in order to evaluate the comparison among MDD patients groups; new visit, non-remission and remission. Additionally, a two-sample *t*-test (or Mann Whitney Wilcoxon test in the case of nonparametric data) was applied in order to determine the significance between the two groups (new visit vs. non-remission, new visit vs. remission, non-remission vs. remission). After determining the significance for ANOVA, Tukey’s multiple comparison method (or Bonferroni’s method in case of nonparametric data) was used for post-hoc group comparisons.

All analyses were two-tailed and calculations were performed using SAS® Version 9.2, SAS institute, Cary, NC, USA.

## Results

### Demographics and clinical profiles

The average age of the three groups did not show a significant difference. The remitted group also showed the lowest HAM-D score and DSSS score among the three groups (Table 1).

**Table 1:** **Demographics of subjects with new visit, remitted and non-remitted MDD**

	New MDD	Remitted MDD	Non-remitted MDD	p	Statistics	Test ^a)^
	(N = 128)	(N = 100)	(N = 109)			
Sex, n(%)						
Male	53(41.4)	43(43.0)	44(40.4)	N.S.		
Female	75(58.6)	57(57.0)	65(59.6)			
Age (years)	42.22 ± 12.36	45.42 ± 11.34	45.12 ± 10.84	N.S.		
Marital status, n(%)						
Single	31(24.2)	20(20.0)	20(18.3)	N.S.		
Married	76(59.4)	64(64.0)	74(67.9)			
Bereavement	5(3.9)	4(4.0)	6(5.5)			
Divorced	12(9.4)	9(9.0)	8(7.3)			
Separated	3(2.3)	3(3.0)	1(0.9)			
Education, n(%)						
Elementary school	16(12.5)	13(13.0)	10(9.2)	N.S.		
Middle school	19(14.8)	14(14.0)	13(11.9)			
High school	37(28.9)	37(37.0)	44(40.4)			
University	49(38.3)	26(26.0)	34(31.2)			
Graduate school	6(4.7)	10(10.0)	8(7.3)			
Onset of MDD (years)	39.67 ± 13.28	42.65 ± 11.75	42.60 ± 11.33	N.S.		
Number of previous depressive episodes	0.82 ± 1.46	1.00 ± 1.24	1.37 ± 2.15	0.0461^b)^	F = 3.11	a = b, b = c, a < c
HAM-D scores	17.48 ± 7.17	4.39 ± 2.19	16.02 ± 5.05	<0.0001^b)^	F = 187.61	b < c = a
DSSS	28.09 ± 12.25	10.15 ± 9.69	27.07 ± 12.93	<0.0001^b)^	F = 77.38	b < c = a
DS	17.16 ± 7.04	5.96 ± 5.92	16.57 ± 7.36	<0.0001^b)^	F = 90.00	b < c = a
SS	10.93 ± 6.16	4.19 ± 4.32	10.48 ± 6.66	<0.0001^b)^	F = 43.91	b < c = a
PS	5.18 ± 3.23	2.20 ± 2.21	5.11 ± 3.62	<0.0001^b)^	F = 31.61	b < c = a

^a)^Tukey’s Post-hoc test.

^b)^Difference between groups (ANOVA).

Note: Denominator of percentage is the number of subjects in the column.

Categorical variables are displayed as the number of subjects (percentage of subjects).

Continuous variables are displayed as mean ± sd.

### Direct cost and indirect cost for previous one month

Regarding direct costs, the non-remitted group showed the highest direct non-medical cost compared to other groups (*χ*^2^ = 12.99, p = 0.0015). When compared to the remitted group, the non-remitted group spent more direct non-medical cost ($19.30 ± 98.82). Direct medical cost did not show a significant difference among the three groups (Table 2).

**Table 2:** **Direct cost for previous one month**

Direct cost for previous one month	New MDD	Remitted MDD	Non-remitted MDD	p	Statistics	Test ^a)^
	(N = 128)	(N = 100)	(N = 109)			
Outpatient cost						
Number of visits to clinics	2.17 ± 1.39(2.00)	2.34 ± 1.51(2.00)	2.91 ± 2.27(2.00)	0.0043^b)^	F = 5.53	a = b < c
Cost of an outpatient treatment($)	77.30 ± 74.43(56.39)	65.94 ± 78.26(50.91)	67.07 ± 58.17(49.09)	N.S.		
Transportation expenses						
Transportation expense for treated MDD	11.18 ± 15.17(7.27)	13.62 ± 31.47(4.18)	15.77 ± 19.09(9.09)	0.0045^c)^	*χ* ^2^ = 10.79	a = b < c
Time spent for transportation (hour)	1.62 ± 1.61(1.00)	1.66 ± 2.01(1.00)	1.91 ± 2.28(1.33)	N.S.		
Supplementary therapy						
Number of subjects	9	9	12			
Average monthly number of supplementary therapy	6.11 ± 8.10(2.00)	9.33 ± 9.99(3.00)	12.25 ± 11.94(7.50)	N.S.		
Average cost of supplementary therapy($)	64.95 ± 108.05(18.18)	51.31 ± 68.00(9.09)	56.35 ± 86.79(9.45)	N.S.		
Type of supplementary therapy, n(%)						
Health supplements	3(33.3)	1(11.1)	2(16.7)	N.S.		
Chinese medicine, acupuncture	1(11.1)	1(11.1)	3(25.0)			
Yoga, meditation, massage	4(44.4)	5(55.6)	7(58.3)			
Physical therapy, exercise	1(11.1)	1(11.1)	0(0.0)			
Etc.	0(0.0)	1(11.1)	0(0.0)			
**Direct medical cost** ^**d)**^	**90.59 ± 84.77(59.09)**	**76.36 ± 81.47(54.55)**	**90.36 ± 85.45(61.99)**	**N.S.**		
**Direct non-medical cost** ^**e)**^	**21.10 ± 55.11(9.09)**	**25.49 ± 52.99(5.64)**	**44.79 ± 126.55(14.55)**	**0.0015** ^**c)**^	*χ* ^2^ = 12.99	**a = b < c**

^a)^Tukey’s Post-hoc test.

^b)^Difference between groups (ANOVA).

^c)^Difference between groups (Kruskal-Wallis test).

^d)^Direct cost = number of outpatient treatment × cost of an outpatient treatment.

^e)^Direct non-medical cost = transportation expense for treated MDD × number of outpatient treatment during the past month + average cost of supplementary therapy × average monthly number of supplementary therapy.

Note: Denominator of percentage is the number of subjects in the column.

Categorical variables are displayed as the number of subjects (percentage of subjects).

Continuous variables are displayed as mean ± standard deviation (median).

Regarding the lost productivity cost, only patients who were currently employed (128, 100 and 109 patients, respectively) were included in the analysis. Actual work hours, absent days and partial missing days due to health problems during the past month were not statistically significantly different among the three groups. LPT due to absenteeism and monthly cost of absenteeism showed a significant difference among the three groups (*χ*^2^ = 6.62, p = 0.0365 and *χ*^2^ = 6.22, p = 0.0445, respectively); however, this significance was lost after using Bonferroni’s correction for post-hoc group comparisons (Table 3).

**Table 3:** **Lost productivity for previous one month**

Lost productive time for previous one month	New MDD	Remitted MDD	Non-remitted MDD	***p***	Statistics	Test ^a)^
	(N = 128)	(N = 100)	(N = 109)			
Actual work (days)	24.20 ± 4.73(25)	23.95 ± 5.41(25)	24.12 ± 5.97(25)	N.S.		
Absent days due to health problems	3.42 ± 6.74(0)	2.83 ± 7.19(0)	4.90 ± 8.69(0)	N.S.		
Partial missing days due to health problems	2.16 ± 4.32(0)	1.69 ± 4.59(0)	2.61 ± 5.70(0)	N.S.		
Other person rating job performance	6.73 ± 2.16(7)	7.28 ± 1.69(7)	6.49 ± 2.33(7)	N.S.		
Self-rated job performance	5.38 ± 2.37(5)	6.93 ± 2.11(7)	5.47 ± 2.49(5)	<0.0001^b)^	*χ* ^2^ = 29.80	
Self-rated job performance compared other person, n (%)						
Extremely better	7(5.5)	11(11.0)	8(7.3)	0.0096^c)^	*χ* ^2^ = 26.33	
Quite better	15(11.7)	15(15.0)	10(9.2)			
Slightly better	10(7.8)	13(13.0)	4(3.7)			
Neither	30(23.4)	30(30.0)	25(22.9)			
Slightly worse	23(18.0)	14(14.0)	23(21.1)			
Quite worse	25(19.5)	10(10.0)	13(11.9)			
Extremely worse	18(14.1)	7(7.0)	26(23.9)			
**LPT due to Absenteeism** ^**e)**^ **(hour)**	**29.59 ± 51.29(2)**	**23.15 ± 53.14(0)**	**38.93 ± 65.69(0)**	**0.0365** ^**b)**^	*χ* ^2^ = 6.62	**a = b = c**
**LPT due to Presenteeism** ^**f)**^ **(hour)**	**69.76 ± 45.71(66)**	**46.19 ± 31.44(45)**	**63.27 ± 47.27(64)**	**0.0004** ^**b)**^	*χ* ^2^ = 15.49	**a = c, a > b, c > b**
Monthly Cost of Absenteeism^g)^ ($)	262.29 ± 438.70(0)	220.25 ± 470.86(0)	449.34 ± 876.63(0)	0.0445^b)^	*χ* ^2^ = 6.22	a = b = c
Monthly Cost of Presenteeism^h)^ ($)	733.43 ± 630.39(559)	517.74 ± 483.64(401)	618.09 ± 563.58(528)	0.0196^b)^	*χ* ^2^ = 7.86	c = b < a
Difference between New and Remitted MDD for LPT due to Absenteeism (hour)	**-**	6.44 ± 52.10	**-**	0.0244^d)^	Z = −2.25	**-**
Difference between New and Remitted MDD for LPT due to Presenteeism (hour)	**-**	23.57 ± 40.13	**-**	<0.0001^d)^	Z = −4.00	**-**
Difference between Non-remitted and Remitted MDD for LPT due to Absenteeism (hour)	-	-	15.77 ± 60.02	0.0205^d)^	Z = −2.32	-
Difference between Non-remitted and Remitted MDD for LPT due to Presenteeism (hour)	-	-	17.08 ± 40.48	0.0140^d)^	Z = −2.46	-
Difference of non-remitted MDD from remitted MDD for Monthly Cost of Absenteeism ($)	-	-	229.11 ± 712.10	0.0205^d)^	Z = −2.32	-
Difference of non-remitted MDD from remitted MDD for Monthly Cost of Presenteeism ($)	-	-	100.35 ± 526.92	0.2347^d)^	Z = −1.19	-
**Total cost ($)**	**1105.55 ± 678.80(990)**	**846.47 ± 698.00(629)**	**1202.36 ± 885.49(951)**	**0.0005** ^**b)**^	*χ* ^2^ = 15.26	**a = c, a > b, c > b**

^a)^Mann Whitney Wilcoxon test for multiple comparison (Bonferroni's correction method).

^b)^Difference between groups (Kruskal-Wallis test).

^c)^Difference between groups (Chi-square test).

^d)^Difference between groups (Mann Whitney Wilcoxon test).

^e)^LPT due to Absenteeism = Absent days due to health problems × 8(hours) + Partial missing days due to health problems × 4(hours).

^f)^LPT due to Presenteeism = [{Actual work (days) × 8(hours)-Absent days due to health problems × 8(hours)-Partial missing days due to health problems × 4(hours)} × {(10-Self-rated job performance)/10}].

^g)^Monthly cost of Absenteeism = LPT of Absenteeism × hourly wage.

^h)^Monthly cost of Presenteeism = LPT of Presenteeism × hourly wage.

Note: Denominator of percentage is the number of subjects in the column.

Categorical variables are displayed as the number of subjects (percentage of subjects).

Continuous variables are displayed as mean ± standard deviation (median).

Regarding presenteeism, the remitted group showed the highest self-rated job performance during the past month (*χ*^2^ = 29.80, p < 0.0001), which is quite similar to the level of rating the job performance of others (6.93 ± 2.11 vs. 7.28 ± 1.69). Self-rated job performance, which compared other people during the past month, showed a significant difference (*χ*^2^ = 26.33, p = 0.0096). The remitted group showed a significantly decreased level in LPT due to presenteeism (69.8 hours for new visit group, 63.3 hours in non-remitted group, and 46.2 hours in remitted group, respectively) and the monthly cost of presenteeism ($733.4 per employee in the new visit group, $618.1 in the non-remitted group, and $ 517.7 in the remitted group).

We also assessed the difference between the non-remitted group and remitted group for LPT due to absenteeism and presenteeism, resulting in 15.8 hours and 17.1 hours per month on average. Further, the monthly cost of absenteeism and presenteeism, which implies the economic benefit we can obtain from achieving remission, was calculated as $229.1 and $100.3 per month per employee, respectively. Although the non-remitted group received treatment at psychiatric clinics similar to the remitted group, it lost 33 more working hours, which is compatible to $332 per month.

We also assessed the correlation between LPT (absenteeism and presenteeism) and symptom severity measured by DSSS and HAM-D. The higher the HAM-D score is, the higher is the LPT due to absenteeism in the new visit group and non-remission group. The correlation coefficients were statistically significant, but numerically low (r = 0.351, p<0.001).

## Discussion

Our results indicated that employees with non-remitted MDD cost more than those of the remitted group both from direct costs as well as lost productivity costs. The results are significant in two aspects. First, we confirmed the tendency of previous studies on productivity and psychiatric disorders, regarding ‘remission’ in depression. The studies suggested that depression has been associated with workplace productivity loss, affecting presenteeism more than absenteeism [22, 23]. Similarly, our results revealed that all three groups of study subjects conveyed more loss from presenteeism than those from absenteeism while showing the least presenteeism in the remitted group (*χ*^2^ = 15.49, p = 0.0004).

Second, these data suggest that psychiatric outpatient treatment itself cannot save substantial costs arising from absenteeism and presenteeism without achieving remission. We can estimate the difference in monthly cost between the non-remission group and remission group as $332, which amounts to 16.3% of the average employment income of $2041 per month in Korea as of 2010.

The remitted group rated their own job performance as 6.90 (out of a possible 10). Hence, no specific rating score is yet defined as a normative value for the general working population. Kessler et al. mentioned that the majority of the workers rated their work performance as over 7, mostly 8 to 9 [14]. Data from previous studies showed that values for the general working population usually fall between 7 and 8, which is a bit higher than that of the remitted group of this study [4, 20].

Given the results of this study, we can assume that the distress among workers with MDD have measurable loss on everyday activities. Doctors may want to relate clinical distress and economic loss for patients with depression and focus on how treatment can reduce the economic loss. Such efforts could improve treatment compliance by enhancing motivation and thus help achieve remission.

This study has some limitations. First, possible selection biases need to be considered. Due to ethical and practical issues, we did not include a randomized control group. Instead, we recruited patients who voluntarily visited a psychiatric clinic in order to get treatment and were working at the moment. However, they may systematically differ from those who do not work. Even though the three groups of subjects were not significantly different in many demographic and work-related variables, the average severity of depression among the subjects of this study can be lower than the depressed patients at the workplace or community. For example, the new visit group and non-remitted group rated their own job performance as 5.4 ± 2.37 and 5.5 ± 2.49, respectively, which are approximately 20% higher than the results from workers with MDD in a previous study [4]. It is also possible that some patients could not maintain their jobs due to MDD, and thus were excluded from this study even if they can be included in the labor force. Therefore, the lost productivity cost from LPT in this study could be underestimated than that in the previous study.

Second, some patients may achieve remission just prior to the time of the assessments. In these cases, the subject may report more severe work productivity loss over the course of the past 4 weeks in spite of reporting few depressive symptoms at the time of measurement.

Last but not the least, we must be careful not to make any crude inference regarding the causality in the cross-sectional study of this type.

## Conclusions

As discussed, residual symptoms and non-remission are known to be associated with impairment in social functioning, low quality of life and extensive health care utilization. Obtaining full remission of depressive symptoms is of crucial importance not only for reducing clinical distress, but also for reducing the socio-economic burden.

## Abbreviations

MDD: Major Depressive Disorder
HAM-D: The 17-item Hamilton Depression Rating Scale
HPQ: The World Health Organization Health and Work Performance Questionnaire
LPT: The lost productive time
DSM-IV-TR: Diagnostic and Statistical Manual of Mental Disorders-IV-Text Revision
DSSS: The Depression and Somatic Symptoms Scale
DS: Depression Subscale
PS: Pain Subscale
SS: Somatic Subscale.
